# Dual-functioning antimalarials that inhibit the chloroquine-resistance transporter

**DOI:** 10.2217/fmb.13.18

**Published:** 2013-03-27

**Authors:** Timothy J Egan, David Kuter

**Affiliations:** 1 Department of Chemistry, University of Cape Town, Private Bag, Rondebosch 7701, South Africa; 1 Department of Chemistry, University of Cape Town, Private Bag, Rondebosch 7701, South Africa. Tel.: +27 21 650 2528; Fax: +27 21 650 5195; timothy.egan@uct.ac.za.

**Keywords:** chloroquine resistance, dual-functional antimalarial, hemozoin, malaria, PfCRT, resistance reverser

## Abstract

Malaria remains a major international health challenge. Resistance to a number of existing drugs and evidence of the emergence of artemisinin resistance has emphasized the need for new antimalarials. A new approach has been the preparation of dual-function compounds that include a chloroquine-like antimalarial group and a group that resembles a chloroquine chemosensitizer. This article reviews the recent discovery of such dual-function antimalarials that are proposed to target both hemozoin formation and the chloroquine resistance transporter, PfCRT. These are discussed in relation to the mechanism of action of 4-aminoquinolines, chloroquine resistance and resistance reversal.

**Figure 1.**
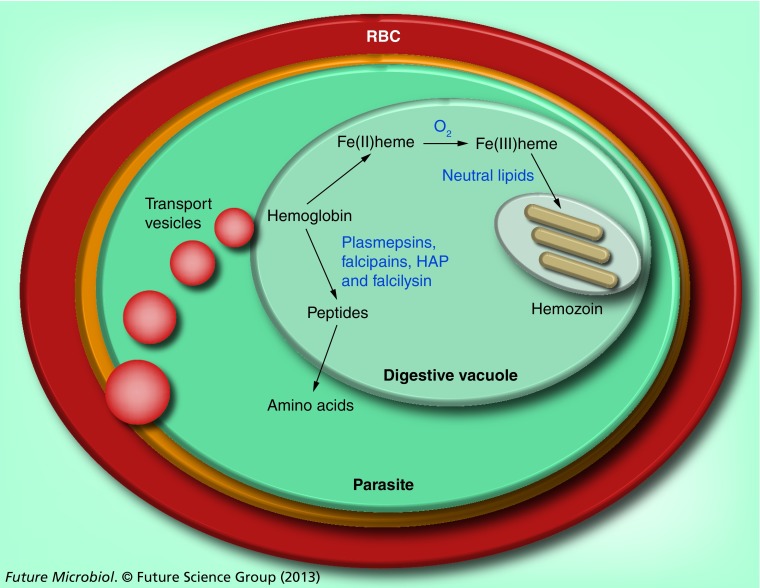
A schematic representation of the process of hemoglobin degradation and hemozoin formation in *Plasmodium falciparum*. RBC cytoplasm is taken up into the malaria parasite via transport vesicles in an endocytotic process and delivered to the acidic digestive vacuole (∼pH5) [86]. Hemoglobin is digested by a series of proteases: plasmepsins, HAP, falcipains and falcilysin [87–89]. The resulting peptides are ultimately hydrolyzed to amino acids. Currently, agreement about the details of this step has not been reached [90–92]. The toxic heme released during hemoglobin digestion is oxidized to the Fe(III) state and then incorporated in less-toxic hemozoin in a process associated with neutral lipids [93,94], represented in the figure by the elliptical structure enclosing the hemozoin crystals. HAP: Histoaspartic protease; RBC: Red blood cell.

**Figure 2.**
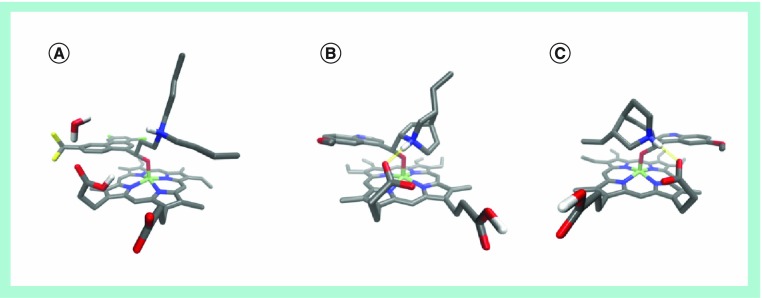
Crystal structures of heme–antimalarial drug complexes. **(A)** Fe(III)heme–halofantrine, **(B)** Fe(III)heme–quinine and **(C)** Fe(III)heme–quinidine. All three drugs possess benzylic alcohol groups that coordinate to the iron center of the heme molecule as deprotonated alkoxides. The aromatic ring lies parallel to the porphyrin ring in a π-stacking arrangement. In the cases of the quinidine and quinine complexes, a charge-assisted hydrogen bond (salt bridge) occurs between one of the heme propionate groups and the protonated quinuclidine tertiary amino group. The structural models shown here were created using data taken from [19,20].

**Figure 3.**
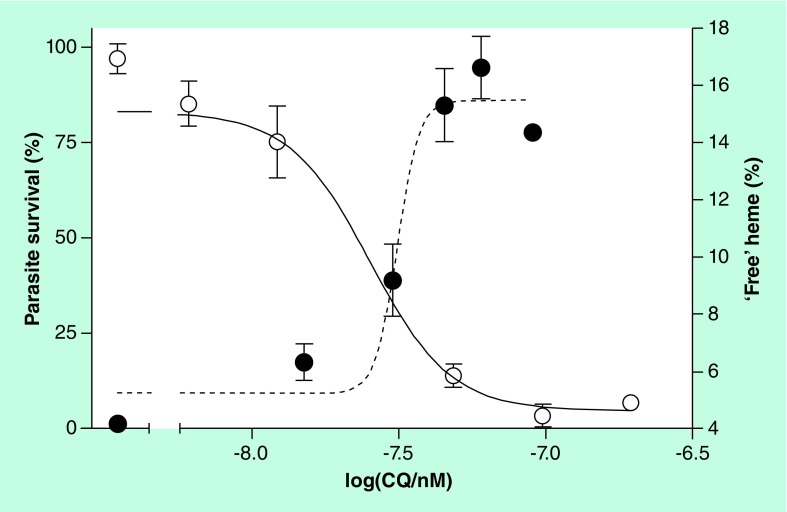
Relationship between detergent-soluble (free) Fe(III)heme and parasite survival in chloroquine-treated *Plasmodium falciparum*. In untreated CQ-sensitive D10 strain parasites, the basal level of free Fe(III)heme is approximately 4% of the total parasite heme. This increases with CQ dose, reaching a maximum of approximately 16% (closed circles). The free Fe(III)heme curve is a mirror image of the parasite survival curve (open circles), crossing close to the IC_50_ value. CQ: Chloroquine. Reproduced with permission from [41] © American Chemical Society (2013).

**Figure 4.**
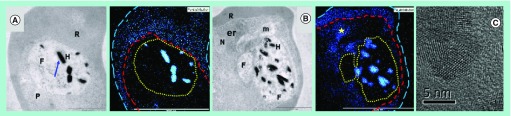
Effects of chloroquine on iron distribution in *Plasmodium falciparum* and on hemozoin crystals. **(A)** Distribution of iron in a control parasite determined by electron energy loss spectroscopy. Left image: transmission electron micrograph. Right image: iron electron spectroscopic image. Since the total iron content of the cell is indistinguishable from the total heme content, the iron distribution coincides with heme distribution. Scale bars = 2 µm. **(B)** Parasite treated with 30 nM chloroquine for 32 h. Note the redistribution of iron into the parasite cytoplasm (star). Scale bars = 2 µm. **(C)** Visible grain boundaries in hemozoin crystals from parasites treated with chloroquine. In these images, the long dashed lines represent the red blood cell membrane, the short dashed lines represent the parasite plasma membrane and the dotted lines represent the digestive vacuole membrane. er: Possible endoplasmic reticulum; F: Digestive vacuole; H: Hemozoin; m: Mitochondrion; N: Nucleus; P: Parasite; R: Red blood cell. Reproduced with permission from [41] © American Chemical Society (2013).

**Figure 5.**
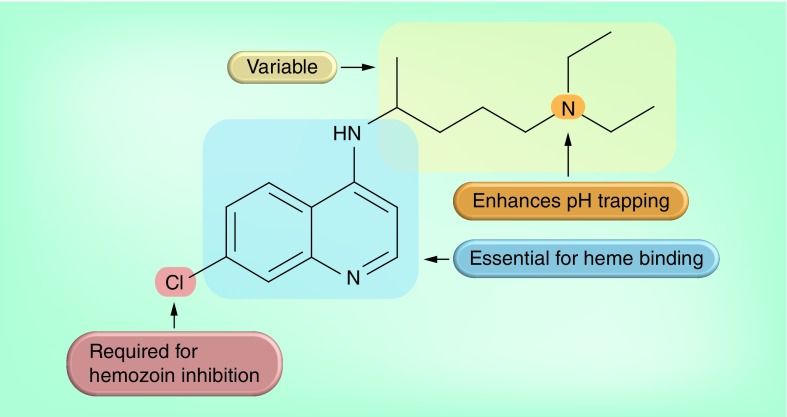
Structure–activity relationships in chloroquine. The 4-aminoquinoline group is the smallest Fe(III)heme-binding fragment [43]. Replacement of the quinoline ring N atom with a CH group abolishes interaction with Fe(III)heme, as well as hemozoin inhibitory activity and biological activity [45]. Replacement of the 4-amino NH group with a CH_2_ group weakens Fe(III)heme binding and also abolishes hemozoin inhibition and biological activity [45]. The Cl atom is necessary for the inhibition of hemozoin formation. It can be replaced with other electron-withdrawing hydrophobic groups [44]. The tertiary N atom in the side chain is important for accumulation in the parasite digestive vacuole through pH trapping, and it usually improves activity [43]. Analogs are known in which this N atom is replaced with a CH, retaining biological activity [42].

**Figure 6.**
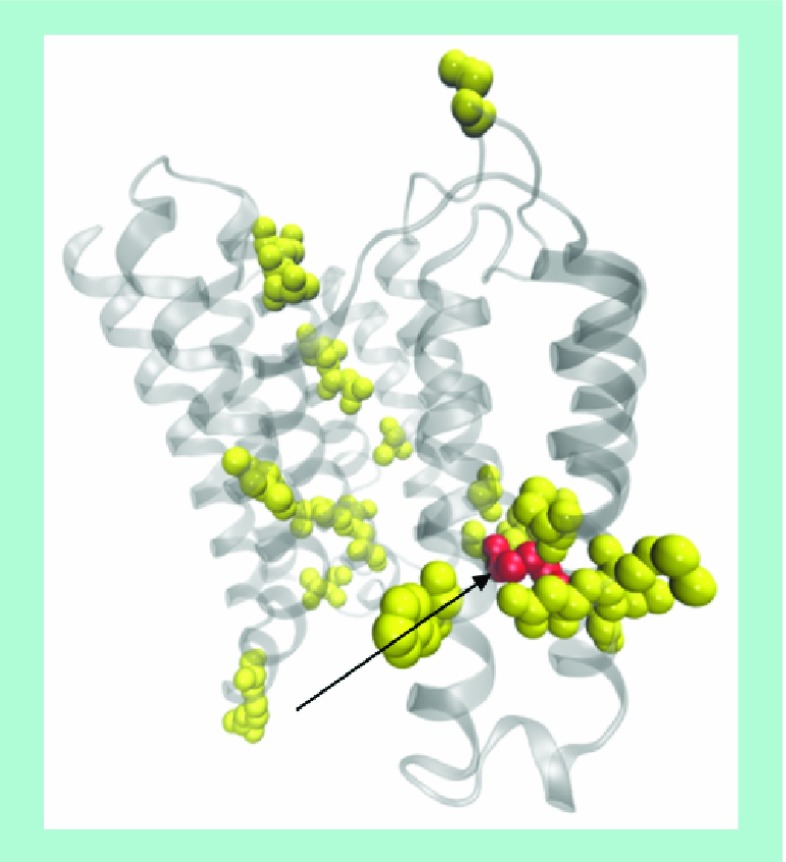
Sites of mutations in PfCRT associated with chloroquine resistance. Sites of known mutations in PfCRT are shown on a structural model of the protein. The model is based on weak homology with *Escherichia coli* multidrug resistance transporter (EmrE). The reliability of this structural model remains untested. Side chains of residues at sites of mutations implicated in chloroquine resistance are shown as space-filling spheres, and K76 is marked by an arrow. The structural model of the protein shown here was created using data taken from [95].

**Figure 7.**
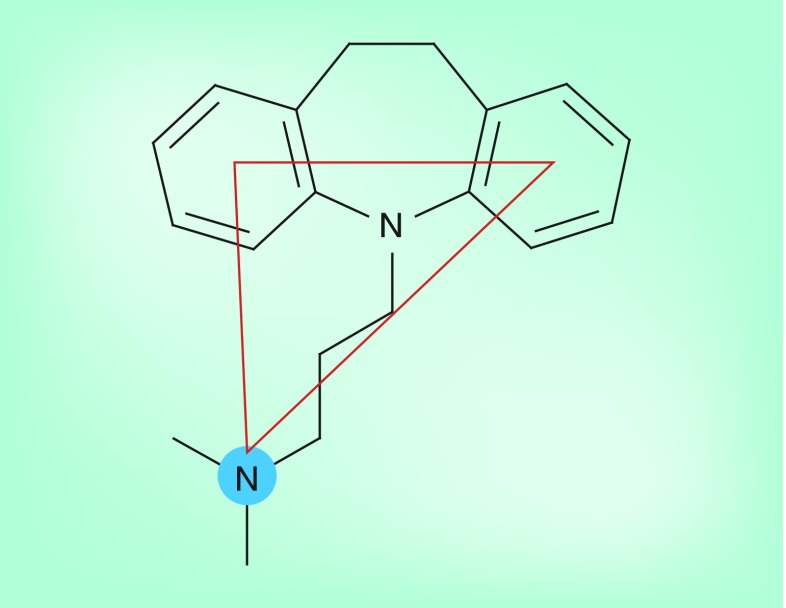
Structure–activity relationships illustrated in the chloroquine chemosensitizer imipramine. The crucial features are two suitably positioned aromatic rings and a hydrogen bond acceptor (or donor in the protonated species). Data taken from [49,74].

**Figure 8.**
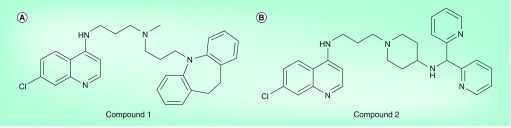
Examples of reversed chloroquines. **(A)** The prototype compound reported by Peyton and coworkers consisting of a 4-amino-7-chloroquinoline group attached to an imipramine molecule **(Compound 1)** [75]. **(B)** A subsequently designed analog **(Compound 2)** with improved water solubility and improved oral bioavailability from the same group [76].

**Figure 9.**
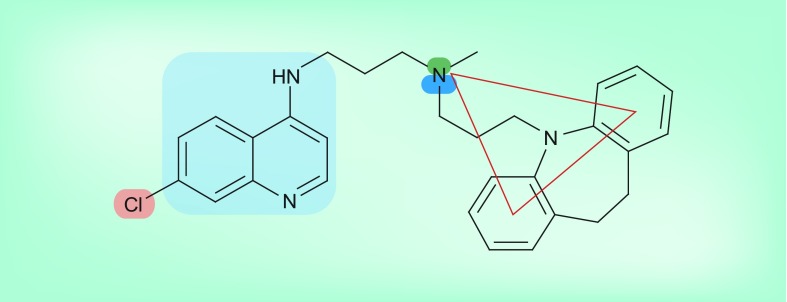
Rationalization of the structure–activity relationships in the reversed chloroquines. The parts of the molecule highlighted are the 4-aminoquinoline moiety required for Fe(III)heme binding, the Cl atom required for hemozoin inhibition, the basic tertiary N atom that aids accumulation in the digestive vacuole (see Figure 5) and the two aromatic rings and hydrogen bond acceptor required for a chloroquine chemosensitizer (see Figure 7).

**Figure 10.**
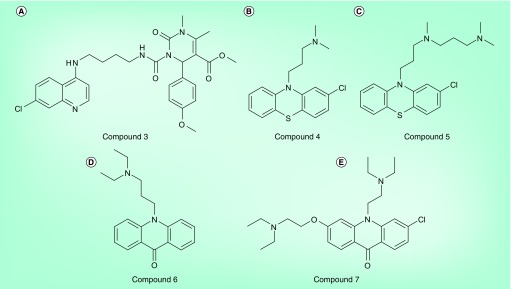
Other reversed chloroquines and related compounds. **(A)** An example of a reversed chloroquine with a dihydropyrimidinone side chain **(Compound 3)** [78]. Dihydropyrimidinones are known calcium channel blockers that reverse drug resistance in cancer cells. Unlike the other reversed chloroquines described here, these molecules lack a basic N atom in the side chain. **(B)** The phenothiazine chlorpromazine **(Compound 4)** is a chloroquine chemosensitizer in chloroquine-resistant parasites and **(C)** can be modified to produce an active antimalarial by the introduction of an additional basic N atom **(Compound 5)** [49]. **(D)** A chloroquine-chemosensitizing molecule **(Compound 6)** built from a related acridone ring [79]. **(E)** A dual-function acridone **(Compound 7)** that both reverses chloroquine resistance and is an active antimalarial [80].

**Figure 11.**
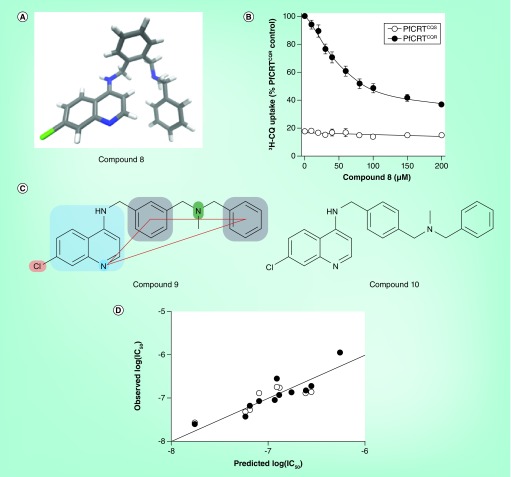
Dibemequines, a new class of antimalarial that inhibits chloroquine transport by PfCRT. **(A)** The crystal structure of the prototype dibemequine compound **(Compound 8)**. **(B)** Inhibition of ^3^H-CQ transport into *Xenopus* oocytes via PfCRT brought about by **Compound 8**. The open circles represent baseline uptake in the presence of PfCRT^CQS^ and the closed circles represent uptake in the presence of the PfCRT^CQR^. **(C)** Analogs of the prototype compound with meta- **(Compound 9)** and para- **(Compound 10)** substituents on the central phenyl ring. The antimalarial and resistance-reversing structural elements are highlighted in the case of **Compound 9**. **(D)** Observed versus predicted activity of the dibemequine series according to the quantitative structure–activity relationship multiple linear correlation equation log(IC_50_) = 0.95log(BHIA_50_) – 0.35 pos – 3.87log(VAR) + 10.24, where ‘BHIA_50_‘ is the IC_50_ for β-hematin formation, ‘pos’ is a structural constant describing the arrangement around the central phenyl ring (2, 3 and 4 for ortho-, meta- and para-substituents as in **Compounds 8, 9 & 10**, respectively) and ‘VAR’ is the predicted vacuolar accumulation ratio, based on p*K_a_* values of the compounds. The equation indicates that activity increases with increasing accumulation in the parasite digestive vacuole, with increasing strength of β-hematin inhibition and with the para-substituted compounds being most active and the ortho-substituted compounds being least active. CQ: Chloroquine; PfCRT^CQR^: Chloroquine-resistant mutant PfCRT; PfCRT^CQS^: Wild-type PfCRT. Reproduced with permission from [81] © American Chemical Society (2011).

## Malaria: a major international health burden

According to the WHO World Malaria Report of 2011, there were 216 million cases of malaria in 2010, with 655,000 deaths, 91% of which occurred in Africa, with 86% of the victims being children under the age of 5 years [1]. Despite a 25% decrease in mortality since 2000, one child still dies of malaria every minute. The disease is caused by four species of protozoan parasite of the genus *Plasmodium* that are specific to humans, namely *Plasmodium falciparum*, *Plasmodium vivax*, *Plasmodium malariae* and *Plasmodium ovale*. A fifth species, *Plasmodium knowlesi*, which primarily infects monkeys, has also been recognized as a zoonotic cause of human malaria in Indonesia [2]. Of these species, *P. falciparum* is the most deadly. All are transmitted by mosquitoes of the genus *Anopheles*.

Control and eradication of malaria relies on a multifaceted strategy. This involves prevention of mosquito bites using screening methods, elimination of mosquitoes and treatment of infected individuals to eliminate the parasite from the host. Large-scale deployment of bed nets [3], reintroduction of DDT in some areas, such as Mozambique [4], and the introduction of artemisinin combination therapy (ACT) have all contributed to the significant progress made in the last decade in fighting malaria [5].

## Drug resistance: an ominous threat

The first highly active synthetic antimalarials introduced after World War II were used either as monotherapies (e.g., chloroquine) or combinations targeting a single pathway (antifolates; e.g., sulfadoxine with pyrimethamine). This strategy eventually led to the emergence and widespread dissemination of drug-resistant parasites. Consequently, starting in the 1990s, new combination therapies in the form of ACTs were introduced. These consist of an artemisinin derivative, such as artesunate, artemether or dihydroartemisinin on the one hand, combined in a fixed dose with a 4-aminoquinoline, aryl methanol or a related derivative such as amodiaquine, lumefantrine (Coartem^®^, Norvatis, Switzerland), mefloquine, piperaquine (Euartesim^®^, MMV, Switzerland) or pyronaridine (Pyramax^®^, MMV) on the other. Currently, these ACTs are highly effective. However, ominous first signs of possible future resistance have begun to appear.

Patients have been identified with prolonged parasite clearance times, resulting in artemisinin treatment failures in western Cambodia and western Thailand [6–8]. This appears to have occurred as a result of the use of artemisinin monotherapy [6]. At the same time, resistance to mefloquine is widespread [9], while some strains of chloroquine-resistant parasite are also cross-resistant to amodiaquine [10]. In addition, evidence suggestive of the possible development of lumefantrine tolerance, which is the first step towards resistance, has begun to emerge in east Africa, where Coartem is widely used [11]. While resistance to ACT may still be some time away, it is prudent that new antimalarials be developed against this possibility. Thus, considerable work continues to find new and improved antimalarials.

## Quinolines & related antimalarials

Chloroquine, the archetypal synthetic quinoline antimalarial, was also historically the most important. Prior to the emergence of resistance, it was highly effective, generally well tolerated at appropriate doses, safe for use in pregnancy and inexpensive. Unfortunately, chloroquine-resistant *P. falciparum* is now very widespread. Nonetheless, this has not led to the complete loss of this class of compound, because resistance is not coupled to the drug target itself. Indeed, the 4-aminoquinoline piperaquine has recently entered the clinic (in combination with dihydroartemisinin) [12], while another 4-aminoquinoline, ferroquine, is in Phase IIb clinical trials [13]. In addition, the 4-aminoquinoline, amodiaquine, and the quinoline methanol, mefloquine, as well as the aryl methanol, lumefantrine, are all currently crucial components of ACT.

These antimalarials are widely believed to act by inhibiting heme detoxification in the malaria parasite, a hypothesis that is best established in the case of chloroquine. The process of hemoglobin digestion and heme detoxification is summarized in Figure 1. Hemozoin formation is the dominant fate of heme released into the digestive vacuole (DV), with at least 95% of the iron present in late trophozoites (32 h into the 48-h blood cycle) present as hemozoin [14]. The parasite also possesses an endogenous cytosolic Fe-superoxide dismutase and imports a host peroxiredoxin from the red blood cell into the parasite cytosol [15,16]. These enzymes remove O_2_^-^ and H_2_O_2_, both of which are probably produced in part during the oxidation of Fe(II)heme.

While it has long been known that antimalarials such as chloroquine and quinine interact directly with Fe(III)heme [17,18], the first crystal structure of a drug–Fe(III)heme complex (that of halofantrine–Fe[III]heme), was only reported in 2008 (Figure 2A) [19]. It was demonstrated that this drug can directly bind to the Fe(III) center of Fe(III)heme via a deprotonated hydroxyl group. Based on this structure as a model, molecular mechanics were used to support a hypothesis that the relative biological activities of the four isomers of quinine against *P. falciparum* could be accounted for on the basis of an additional interaction: a charge-assisted hydrogen bond between a protonated amine nitrogen atom in the side chain of the drug and one of the heme propionate groups. This interaction has recently been confirmed in crystal structures of quinine–Fe(III)heme and quinidine–Fe(III)heme (Figure 2B & 2C) [20].

By contrast, the structures of 4-aminoquinoline–Fe(III)heme complexes remain less well understood, despite various efforts to use nuclear magnetic resonance techniques to elucidate them [21–29]. In addition, the precise relationship between heme binding and hemozoin inhibition remains unclear. Early postulates involved stoichiometric solution complexes of antimalarials with Fe(III)heme [30] and included suggestions that drugs act by increasing Fe(III)heme solubility, thus preventing aggregation [30,31]. More recently, however, an alternative suggestion by Buller *et al.* has enjoyed considerable attention [32]. These authors, among others, have suggested that this class of drug can dock into the fastest-growing face of the hemozoin crystal, as well as inhibit growth of some of the other faces [32–35]. This hypothesis has been able to mathematically account for the effects of chloroquine and quinidine on the kinetics of synthetic hemozoin (β-hematin) formation [36]. In addition, it can also explain how substoichiometric quantities of a drug can inhibit hemozoin formation and provides a well-defined binding site for these compounds. To date, however, this model does not appear to have been directly used to try to design new inhibitors using a rational approach, probably because conventional drug-docking programs are not able to handle crystal surfaces well.

Despite ample evidence that quinoline and related antimalarials inhibit β-hematin formation under abiotic and biomimetic conditions, direct evidence of inhibition of hemozoin formation in the parasite itself is much more sparse. Chloroquine and, more recently, ruthenoquine, an analog of the 4-aminoquinoline ferroquine in which the ferrocene moiety is replaced by ruthenocene, have been shown to accumulate in the parasite DV in close proximity to hemozoin [37,38]. Smaller hemozoin crystals and a premature halt in their growth within the parasite DV has been observed in the presence of chloroquine [39]. Finally, at 120 nM and 12-h incubation times, chloroquine causes a build-up of transport vesicles in the parasite, which contain undigested Hb [40], indicating that the endocytotic feeding process is inhibited. Recently, a fractionation strategy has been applied together with electron spectroscopic imaging to chloroquine-treated *P. falciparum* [41]. This has clearly shown a dose-dependent increase in free Fe(III)heme occurring together with the decrease in hemozoin. Undigested Hb only seems to appear at higher doses of chloroquine, and the levels of free Fe(III)heme are closely correlated with the parasite survival curve (Figure 3). Electron spectroscopic imaging using electron energy loss spectroscopy clearly showed a translocation of iron to the parasite cytoplasm (Figure 4A & 4B). Since virtually all of the iron present is heme iron and there is little undigested Hb at the dose used, this is likely to be the location of the free Fe(III)heme. There is also evidence that chloroquine causes a disruption in the growth of the hemozoin crystals, which show evidence of a mosaic structure with grain boundaries (Figure 4C). Thus, in combination with earlier work, this study provided strong evidence that chloroquine does indeed act by inhibiting hemozoin formation in the parasite. Preliminary evidence also showed an increase in free Fe(III)heme and a decrease in hemozoin at 2.5× IC_50_ for a number of other drugs, including amodiaquine, mefloquine and lumefantrine, as well as artesunate. However, it must be emphasized that further work would be required to substantiate whether the observations made with these other drugs are causal or the effect of inhibiting other targets.

Despite ongoing efforts to understand the mechanism of action of 4-aminoquinolines and the structures of their complexes with Fe(III)heme, the existence of structure–activity relationship data has substantially aided the task of designing new compounds in this class. Four studies in the late 1990s and early 2000s provided a detailed structure–activity model for the 4-aminoquinolines [42–45]. This is summarized in Figure 5. Further studies have revealed that alterations to the alkyl side chain of the quinoline can abolish cross-resistance with chloroquine [46]. Indeed, the structure of the side chain appears to be the primary determinant of resistance, with the quinoline ring itself having, at most, a minor role in cross-resistance [47–49]. This has encouraged the design of new quinoline antimalarials with the aim of overcoming resistance.

## Chloroquine resistance & PfCRT

As mentioned above, *P. falciparum* that are resistant to chloroquine or other quinolines exhibit no known changes in the process of heme detoxification. Rather, resistance arises from mutations and changes in expression levels of membrane proteins located in the DV membrane. The principal determinants of chloroquine resistance are mutant forms of a protein known as PfCRT [50,51]. A second protein, PfMDR1, has also been implicated in resistance to quinoline antimalarials [52,53]. Mutations in this protein are associated with mefloquine resistance in field isolates, and an increased copy number has previously been associated with decreased sensitivity to quinine. PfMDR1 is not thought to be directly responsible for chloroquine resistance, but mutant forms of this protein can affect chloroquine sensitivity in the presence of mutant forms of PfCRT [54], and there is evidence of a complex interaction between PfCRT and PfMDR1 [55]. In addition, PfMDR1 has recently been implicated in the transport of chloroquine and other quinolines into the parasite DV [56] and has subsequently also been shown to bind to a selection of these drugs [57]. Furthermore, evidence from a cross of two different drug-resistant strains (GB4 and 7G8) has indicated that quinolines actually inhibit transport of the natural substrates of PfCRT and PGH-1 (the protein encoded by *PfMDR1*) [58]. A third protein, PfMRP1, located in the parasite plasma membrane, has also been suggested to play a role in chloroquine resistance, but in this case, the evidence remains uncertain [59,60]. Notwithstanding the role of these other membrane proteins, a PfCRT mutation is accepted to be the major factor involved in chloroquine resistance.

PfCRT is predicted to be an integral membrane protein localized to the DV membrane. It is believed to be a member of the drug metabolite transporter family of proteins [61]. Chloroquine-resistant strains of the parasite exhibit several mutations in this protein (Figure 6) [62], but all naturally occurring chloroquine-resistant mutants exhibit one crucial mutation, that of Lys-76 to Thr-76 (K76T). The additional mutations to K76T are thought to counteract the loss of function that would occur in the case of the K76T mutation alone [63]. Studies conducted using *Xenopus laevis* oocytes injected with mRNA encoding PfCRT have convincingly shown that this protein transports chloroquine. These findings support the hypothesis that decreased activity of chloroquine stems from its extrusion by PfCRT from the DV. This effectively lowers the concentration of chloroquine in this organelle, thus permitting hemozoin formation to resume unhindered [64]. Chloroquine is also thought to bind to PfCRT, and a possible site of interaction has been proposed based on photoaffinity labeling [65]. Several recent and comprehensive reviews covering PfCRT and chloroquine resistance are available, and readers are encouraged to consult them for in-depth discussion [62,66–68].

It has been known for two and a half decades that verapamil, a calcium channel blocker, can restore the activity of chloroquine in several resistant laboratory strains of *P. falciparum* [69,70]. Subsequent to this initial discovery, many other compounds have been found to have similar chemosensitizing properties. These include other calcium channel blockers, including analogs of verapamil and nifedipine; dibenzazepines and their analogs, which include imipramine; phenothiazines; dihydroanthracenes; dibenzylmethylamines (dibemethins); plant-derived alkaloids; and others, including primaquine [71]. A common feature of nearly all of these compounds is a basic amino group that is expected to be protonated at the pH of the parasite DV. These compounds are believed to act by inhibiting chloroquine transport by PfCRT, and this has been directly demonstrated in the case of verapamil, primaquine and several dibemethins in the *Xenopus* oocyte system [64,72]. It is not known whether these compounds act as competitive or noncompetitive inhibitors of chloroquine transport.

Two significant quantitative structure–activity relationship studies have been carried out on chloroquine chemosensitizers. One exclusively investigated a series of dihydroanthracene derivatives with rigid bicyclic structures [73]. Based on this study, the authors suggested that these molecules interact with a serine (or threonine) and an aspartate (or glutamate) side chain in PfCRT, which are separated by 9.2 Å. In a second study, which has turned out to be more influential, a series of imipramine analogs were investigated, leading to a pharmacophore model consisting of two suitably positioned aromatic groups and a weak base amino group (Figure 7) [74]. The latter study has underpinned the design of a new type of antimalarial that incorporates the features required for both an active antimalarial and a resistance-reversing chemosensitizer. These dual-function compounds, consisting of so-called ‘reversed chloroquines’ and related compounds, are the subject of the remainder of this review.

## Reversed chloroquines & related compounds

Dual-function antimalarials with both chloroquine-like activity (hemozoin inhibition) and resistance-reversing activity form a relatively new class of compounds. The first example, a so-called reversed chloroquine **(Compound 1)**, was reported in 2006 [75]. This consisted of a 4-amino-7-chloroquinoline moiety linked to the N atom of imipramine via a three-carbon alkyl linker group (Figure 8A). **Compound 1** was shown to be active *in vitro* against both the chloroquine-sensitive D6 and chloroquine-resistant Dd2 strains of *P. falciparum*. It was also shown to associate with Fe(III)heme, both at pH 5.7 and 7, with log association constant (log*K*) values of 5.48 (comparable to chloroquine, with values of 5.48 and 6.00, respectively, at these two pH values). However, **Compound 1** itself was not considered suitable for further drug development owing to its high lipophilicity (log distribution constant [clogP] = 8.9). In a follow-up study, Peyton and coworkers investigated the effects of the linker and head groups in the activity of a range of reversed chloroquines [76]. In one study, the head group was altered to a diphenylamine or dibenzylamine, while the aliphatic portion of the side chain and linker chain were varied in length in an effort to reduce lipophilicity [76]. All of these compounds demonstrated strong activity against parasites cultured *in vitro* (IC_50_ <120 nM), with only minor differences between chloroquine-sensitive and -resistant D6 and Dd2 strains. Larger differences were seen in cytotoxicity, with molecules possessing amide or piperazine linkers exhibiting the lowest toxicity. Nonetheless, all of the compounds tested exhibited well over 100-fold selectivity against malaria parasites. Closer scrutiny of the IC_50_ values showed that a dibenzylamine head group usually gave rise to a more active compound than the corresponding compound with a diphenylamine head group, and that replacement of the linear diamine linker with a piperazine group also generally decreased activity. On the other hand, introduction of an amide head group was tolerated and permitted a decrease in clogP to values comparable to chloroquine.

In a subsequent and more comprehensive study, this group made further variations to this chemotype, replacing the diphenyl- or dibenzyl-amine head group with others such as benzhydryl, adamantyl, triphenylmethyl and pyridine-2-yl methyl groups [77]. Benzhydryl head groups with substituents were also investigated. Interestingly, the variations in head group had a relatively small influence on activity against either the D6 chloroquine-sensitive or Dd2 chloroquine-resistant strains of the parasite, but considerably more variation was observed in activity against the 7G8 chloroquine-resistant strain. Indeed, in many of the derivatives, activity against the 7G8 strain was four- to five-fold weaker than against the D6 or Dd2 strains. This is notable in view of the fact that the 7G8 strain, which originates from South America, differs from the Old World D6 and Dd2 strains in as much as verapamil has only weak chemosensitizing activity on it. This perhaps supports the proposal that the activities of these compounds relates to the presence of a resistance-reversing pharmacophore. Of particular note, however, is a derivative, **Compound 2**, in which the phenyl rings in the head group were replaced with ortho-pyridyl groups (Figure 8B) [77]. This compound was equipotent against all three of the tested strains and is substantially less lipophilic (clogP = 3.6) than the other compounds, a factor that is important in improving solubility and potentially lowering systemic toxicity. This compound represents a potential lead compound for further development. Indeed, it was found to possess good oral activity in the *Plasmodium berghei* mouse model of malaria, with four doses at 30 mg/kg reducing parasitemia by more than 99% and curing two out of three treated mice.

A selection of these reversed chloroquines were investigated for their Fe(III)heme binding and β-hematin inhibiting activities. They were found to exhibit dissociation constant values (*K*_d_) ranging from 8.6 to 1.0 µM (log*K* = 5.1–6.0), similar to that of chloroquine with a *K*_d_ of 4.0 µM (log*K* = 5.4). The IC_50_ values for β-hematin inhibition (1.6–14 µM) were lower than that of chloroquine (24 µM), and a significant (R^2^ = 0.66) correlation between β-hematin inhibitory IC_50_ and *in vitro* antimalarial activity against *P. falciparum* was observed. Finally, it has also been demonstrated that these compounds could inhibit β-hematin formation and decrease hemozoin formation within the parasite cell. The most active compound was also a more potent hemozoin inhibitor than **Compound 1** [77].

Consideration of the structures of these reversed chloroquines in light of known structure–activity relationships for active hemozoin-inhibiting quinolines, as well as resistance-reversing chemosensitizers, permits rationalization of their structure–activity relationships (Figure 9). This approach was subsequently used in the design of other resistance-reversing antimalarials.

### Dihydropyrimidinone-containing reversed chloroquines & dual-function acridones

Other studies have built on the concept of reversed chloroquines. Dihydropyrimidinones are a well-known class of calcium channel blockers. Similar to verapamil, they are capable of chemosensitizing multidrug-resistant cancer cells. These molecules have been attached to the 4-amino-7-chloroquinoline structure to produce a series of compounds (e.g., **Compound 3** in Figure 10A) with strong activity against chloroquine-resistant and -sensitive parasites [78]. Interestingly, these compounds do not have basic N atoms in the side chain and therefore do not conform to the more commonly observed structure–activity relationship model.

Tricyclic heteroaromatics, especially phenothiazines such as chlorpromazine **(Compound 4)**, are well-known chloroquine chemosensitizers. Furthermore, it has been demonstrated that chlorpromazine (Figure 10B) can be modified to produce an analog **(Compound 5)** with antimalarial activity by the introduction of an additional basic amino group (Figure 10C) [49]. Kelly *et al.* later showed that the related tricyclic aromatic acridones (e.g., **Compound 6**) also exhibit chloroquine chemosensitizing activity (Figure 10D) [79]. Subsequently, these authors went on to introduce a further weak base-containing side chain onto this scaffold to produce a dual-function compound **(Compound 7)** that exhibited antimalarial activity (Figure 10E) [80]. This compound was shown to accumulate in the parasite DV by confocal fluorescence microscopy and to inhibit hemozoin formation. Isobolograms were used to demonstrate that it exhibited an additive relationship when mixed with chloroquine in a chloroquine-sensitive *P. falciparum* strain (D6), but was synergistic in the chloroquine-resistant Dd2 strain. This observation is consistent with that expected if the compound acts as a chemosensitizer in chloroquine-resistant parasites and also acts as an antimalarial in a manner similar to chloroquine. This compound exhibited excellent activity against chloroquine-sensitive and -resistant parasites and was active *in vivo*.

### Dibemequines

A recent addition to the class of dual-function quinolines that have resistance-reversing activity is the dibemequines [81], consisting of a 4-amino-7-chloroquinoline with a dibemethin (dibenzylmethylamine) side chain. These were designed to fulfill the structure–activity relationship criteria of both an active hemozoin-inhibiting quinoline antimalarial and a chloroquine chemosensitizer (Figure 11). The dibemethin side chains themselves were found to reverse chloroquine resistance and to inhibit chloroquine transport by PfCRT [72]. The crystal structure of the prototype dibemequine **(Compound 8)** demonstrated the structural requirements for a resistance reverser, with the quinoline group folded around to establish an approximately triangular relationship between the quinoline N atom and the two phenyl groups of the dibemethin side chain.

This compound was shown to directly inhibit chloroquine transport by PfCRT in the *Xenopus* oocyte model (Figure 11B). Two analogs **(Compounds 9 & 10)** were also shown to exhibit such activity (Figure 11C). These represent the first blood-stage antimalarially active compounds for which direct evidence of the inhibition of chloroquine transport by PfCRT has been provided. Isobologram analysis of **Compound 10** revealed an additive relationship with chloroquine in the chloroquine-sensitive D10 strain of parasite, but a synergistic relationship in the chloroquine-resistant Dd2 strain. This strongly supported the hypothesis that this class of compound inhibits PfCRT in the parasite under conditions in which it inhibits parasite growth. These compounds were also shown to inhibit β-hematin formation, thus also supporting the hypothesis that their activity against malaria parasites is linked to inhibition of hemozoin formation. Indeed, their biological activity against *P. falciparum* cultured *in vitro* was found to be correlated with β-hematin inhibitory activity, albeit in combination with the predicted DV accumulation ratio and substitution pattern in the dibemethin side chain, with F = 9.70 > F^0.95^ = 8.45 (Figure 11D).

The dibemequines tested for cytotoxicity, which included the three most active derivatives, were found to have low cytotoxicity, with selectivity indices well above 1000. The whole series was found to show little cross-resistance with chloroquine in the K1 strain of chloroquine-resistant parasite. **Compound 10** was also highly active against the K1, Dd2, W2 and RSA11 strains of chloroquine-resistant parasites, with resistance indices below 2. The three prototype compounds **(Compounds 8, 9 & 10)** also had good *in vivo* antimalarial activity in the *P. berghei* mouse malaria model. Two of the compounds were curative when using three or four oral doses at 100 mg/kg, with parasites being undetectable 30 days after treatment and with 100% of the mice surviving. This series of compounds again illustrates the potential of the dual-function approach to chloroquine resistance-reversing antimalarially active compounds. In comparison with the initially reported reversed chloroquine compounds, the clogP values of this series were considerably lower (5.42–6.19) and were comparable to chloroquine (clogP = 5.1). However, less hydrophobic compounds would still be more desirable and represent a priority for any further development of this series.

## Future perspective & challenges

Several dual-function quinolines designed to exhibit both antimalarial and chloroquine resistance-reversal properties have been made that exhibit good *in vivo* activity, have low cytotoxicity and have been shown by isobologram analysis to work synergistically with chloroquine, supporting the hypothesis that they do indeed inhibit chloroquine transport by PfCRT. In addition, in at least one group, the dibemequines, inhibition of the transport of chloroquine by PfCRT in the *Xenopus* oocyte has been directly demonstrated. In a review, Peyton has also reported that similar activity has been observed, but not yet published in the case of the reversed chloroquines [82]. These compounds have been shown to exhibit strong *in vitro* antimalarial activity against a substantial number of chloroquine-sensitive and -resistant parasite strains, with no significant cross-resistance with chloroquine. Thus, in many respects, these compounds have excellent properties for further development. However, there are some serious obstacles that will need to be overcome.

Weak base compounds, particularly hydrophobic weak bases, frequently exhibit inhibitory activity against hERG, a potassium channel found in cardiac muscle. This can lead to prolonged QT intervals and potentially fatal heart arrhythmias [83]. Indeed, in the case of at least one antimalarial drug, halofantrine, this problem has actually been encountered clinically [84]. Unfortunately, hERG toxicity is difficult to predict with certainty. Nonetheless, Gleeson has carried out a principal components analysis of approximately 30,000 compounds for which absorption, distribution, metabolism, excretion and toxicity data had been collected at GlaxoSmithKline and found that while there is a greater potential for hERG liability in basic molecules than in acidic or neutral ones, the liability is generally much reduced if the compound has a molecular weight below 400 and a calculated cLogP below 4 [85]. Indeed, this also improves druggability in a number of other ways. For example, it improves solubility, permeability and volume of distribution, decreases protein binding and lowers the potential of the compound to inhibit cytochrome P450. As noted above, Peyton and coworkers have already produced reversed chloroquines with much improved cLogP values [77]. A dibemequine analog has also been produced with a cLogP well below 4 and a molecular weight below 400 [Egan TJ *et al.*, Unpublished Data]. While it has not yet been demonstrated that any of these compounds actually have reduced hERG liability, it does at least suggest that improvement is possible with this class of compound.

A second challenge relates to the mechanism of inhibition in the mutant PfCRT. It is not currently known whether these compounds inhibit chloroquine transport competitively or noncompetitively. They could bring about inhibition either by themselves being transported in preference to chloroquine or by binding to PfCRT and blocking chloroquine transport. The latter seems more likely, since one would expect resistant parasites to be resistant to the dual-function molecule if it were transported out of the DV more efficiently than chloroquine. If used as monotherapies, these compounds might have an increased risk of rapid development of resistance because of their ability to bind to chloroquine-resistant mutants of PfCRT. It is possible that point mutations may then lead from binding to transport. This potential problem would likely be lessened by use of combination therapy. In addition, with the exception of one group of reversed chloroquines, the activities of these compounds against South American strains such as 7G8, with reduced sensitivity to verapamil resistance reversal, have not been explored. Lack of activity against such strains is potentially a risk factor for these compounds.

The final and probably most difficult hurdle is not a technical one. Currently, as noted earlier, a number of new quinolines and related compounds are in development. In addition, there are several such compounds in current use. Obtaining the necessary support to develop yet another set of quinoline compounds under these circumstances is likely to be a major challenge and probably represents the single biggest hurdle to further development of this class of compound.

Despite the challenges noted above, the dual-function antimalarials that act against both hemozoin formation in the parasite and chloroquine-resistant mutants of PfCRT represent a unique series of molecules with considerable potential as antimalarials. Combinations with existing quinolines and related compounds, including chloroquine, amodiaquine and quinine, which are all thought to be transported by PfCRT, are likely to result in considerably enhanced activities in drug-resistant strains. In common with the other quinolines, they are readily amenable to synthesis and are potentially cheap to produce. In this regard, further investigation of this class of compound is well justified.

**Executive summary**

***Malaria & resistance to antimalarials***

- ▪ Malaria is responsible for the deaths of over 600,000 people a year, approximately 90% of them in Africa.
- ▪ Resistance to existing drugs and evidence of delayed parasite clearance with artemisinins, which is currently the most important class of antimalarial, is a major cause for concern.
- ▪ Continued discovery of new antimalarials is an important strategy.

***Quinoline antimalarials & their mechanism of action***

- ▪ Historically, the quinolines have been one of the most important classes of antimalarial drug.
- ▪ They are known to interact with Fe(III)heme. Recently, crystal structures of the Fe(III)heme complexes of the quinoline methanols, quinine and quinidine and the related aryl methanol, halofantrine, have been reported.
- ▪ Chloroquine is believed to act by inhibiting the incorporation of Fe(III)heme, released when the parasite digests host hemoglobin, into hemozoin, an insoluble crystalline form of Fe(III)heme.
- ▪ Recent work has shown that chloroquine causes a dose-dependent increase in free Fe(III)heme and a decrease in hemozoin in treated parasites that is correlated with parasite survival. In addition, chloroquine treatment has been shown to redistribute heme into the parasite cytoplasm and disrupt the hemozoin crystal lattice.

***Chloroquine resistance, PfCRT & chloroquine chemosensitizers***

- ▪ Chloroquine resistance is now widespread.
- ▪ Such resistance is largely attributable to mutations in a protein, PfCRT, found in the membrane of the digestive vacuole where hemoglobin digestion takes place.
- ▪ A mutant form of PfCRT from chloroquine-resistant parasites has been shown to directly transport chloroquine in *Xenopus laevis* oocytes.
- ▪ A variety of compounds have been discovered that chemosensitize chloroquine-resistant parasites to chloroquine. These are often referred to as resistance reversers.

***Reversed chloroquines & dual-function compounds that inhibit parasite growth & chloroquine transport by PfCRT***

- ▪ Combination of a 4-amino-7-chloroquinoline with an imipramine-like group led to the first example of a putative dual-function ‘reversed chloroquine’.
- ▪ Subsequent work has led to improvements in water solubility and oral bioavailability of this class of compound.
- ▪ Reversed chloroquines have been shown to interact with Fe(III)heme and inhibit parasite hemozoin formation.
- ▪ New reversed chloroquine-like molecules have been discovered, including dual-function acridones, reversed chloroquines containing a dihydropyrimidinone group and dibemequines.
- ▪ Dibemequines inhibit β-hematin formation and have been shown to directly inhibit chloroquine transport by PfCRT.
- ▪ The dibemequines have been shown to maintain activity against a range of chloroquine-resistant parasite strains, to exhibit little cytotoxicity and to be curative for mouse malaria.

***Future perspective***

- ▪ Dual-function antimalarials that inhibit chloroquine transport by PfCRT are innovative compounds that have been shown to have good activity, including oral activity in mice.
- ▪ Obstacles to future development do, however, exist. These include potential for hERG toxicity, the possibility of increased potential for the development of resistance resulting from interaction with PfCRT and the need to compete with numerous other quinoline antimalarials.
